# Workplace learning: the bidirectional relationship between stress and self-regulated learning in undergraduates

**DOI:** 10.1186/s12909-024-06021-w

**Published:** 2024-09-27

**Authors:** Stephan Marsch, Takuya Yanagida, Evelyn Steinberg

**Affiliations:** Vice-rectorate for Study Affairs and Clinical Veterinary Medicine, University of Veterinary Medicine Vienna, Vienna, Austria

**Keywords:** Undergraduates, Students, Veterinary, Workplace, Clinical training, Self-regulated learning, Stress, Longitudinal interplay, Cross-lagged panel, Multilevel vector autoregressive models, CLP, M-VAR

## Abstract

The present study focused on the relationships between various aspects of self-regulated learning (SRL) and stress among undergraduate health science students in workplace settings. Although both constructs are associated with academic achievement it is still unclear how they influence each other. Employing a longitudinal diary design, the aim of the present study was to examine whether perceived stress in the previous week impacts SRL-aspects in the current week and, conversely, whether SRL-aspects in the previous week impacts stress in the current week. Subjects were 192 undergraduate health sciences students in their workplace placements. SRL-aspects and stress were assessed using scales and previously tested single-item measures. The 21 SRL-aspects used in this study included cognition (learning strategies), motivation, emotion, perception of the learning environment, and regulation of these areas on a metalevel (monitoring and control). Data collected over 15 weeks were analyzed using multilevel vector autoregressive models, with the data nested within weeks and one model dedicated to each SRL-aspect and its relationship with stress. Among the 21 path estimates assessing the impact of prior stress on individual SRL-aspects, 10 were statistically significant. For individual SRL-aspects impacting stress, 7 out of 21 paths were statistically significant (*p* < .05). Notably, no model showed statistical significance of effects in both directions. Except for two results, cross-lagged relationships were negative, indicating that better SRL-aspects from the previous week resulted in reduced stress in the current week and vice versa. The effects for the cross-lagged paths from SRL-aspects to stress were predominantly of medium size, whereas the influence of stress on individual SRL-aspects was predominantly small. The present study highlights a potentially causal and mostly negative relationship between stress and various aspects of SRL, but also that the individual relationships require differentiated consideration. The results can be used to develop targeted interventions in the practical part of the training of health science students to reduce stress and improve specific aspects of SRL. Furthermore, these findings underscore assumptions regarding connections between anxiety and increased stress, negative relationships between stress and motivation, and the importance of effective time management strategies for stress reduction.

**Supplementary Information:**

The online version contains supplementary material available at 10.1186/s12909-024-06021-w.

## Introduction

In health science education curricula, undergraduate students must regulate their learning not only within classrooms but also in real-world workplace contexts, particularly during their clinical practical semester or year [1]. This phase represents most students’ first extended exposure to professional environments and is often perceived as particularly stressful and challenging [2, 3]. However, although past studies have shown that both, stress and self-regulated learning (SRL), influence the academic success of undergraduate health sciences students [4, 5], comprehensive studies investigating how they influence each other dynamically are missing. A theoretical framework to understand this interaction is cognitive load theory (CLT; [6]), which suggests that stress can increase extraneous cognitive load, thereby reducing cognitive resources available for SRL activities such as goal-setting and monitoring [6, 7]. Conversely, effective SRL can help mitigate stress by improving academic outcomes [2, 8] and enhancing time management [9]. Consequently, the present study sought to investigate the longitudinal bidirectional relationships between aspects of undergraduate students’ SRL and stress in workplace environments. By investigating this link, this research aims to provide a better understanding of the challenges faced by health sciences students in a clinical setting. The findings are expected to contribute to a more comprehensive understanding of how stress influences SRL processes and, conversely, how effective self-regulation can potentially mitigate the adverse effects of stress, particularly in workplace and higher education settings.

### SRL in the workplace

SRL and workplace learning are related but distinct concepts [10, 11]. SRL is a dynamic process in which individuals take an active role in their own learning by setting goals, monitoring their progress, and adapting their strategies to achieve those goals [12–14]. SRL involves a range of strategies‒including cognitive, metacognitive, and motivational‒that learners employ to effectively acquire and retain knowledge. SRL not only fosters greater academic achievement in students [15, 16] but also cultivates lifelong skills for independent, self-directed learning [17]. Workplace learning, on the other hand, generally refers to the context in which learning takes place within a work environment [10]. However, certain forms of workplace learning can be considered SRL when they involve similar characteristics, such as goal-setting, self-monitoring, and strategy adaptation [11]. Although most SRL literature addresses learning in classroom settings, SRL also plays a major role during learning at the workplace, when acquiring practical skills [18–20]. In this demanding environment, students face the challenge of balancing new responsibilities, such as patient interaction and treatment, while achieving their learning objectives [5]. In this context, SRL can help students attain more meaningful and sustainable learning outcomes. For example, previous studies have shown that SRL is positively associated with academic achievement and clinical skills in the workplace ([21–24]; see [2] for a scoping review on the topic). In this paper, we focus on conceptualizing learning processes through the lens of SRL, with the workplace environment serving as the context where these processes occur.

Drawing from Pintrich’s [14] component-based approach to SRL, Steinberg and colleagues [19] apply an educational psychology perspective to SRL in workplace settings. They describe workplace learning as operating on two distinct levels: the learning process level and the metalevel. This distinction, as previously established by Wirth and colleagues [25], suggests that the learning process level refers to the learning process itself, while the metalevel refers to the students’ regulation of the learning process to reach the desired learning outcome. Steinberg et al. highlight four key areas of SRL in the workplace: cognition, motivation, emotion and context (i.e., perception of the learning environment [19, 26–28]. At the learning process level, the optimal learner employs cognitive and proximal metacognitive learning strategies (proximal means regulating the professional medical activity, not the learning process itself), is motivated, experiences positive emotions during learning and perceives his or her learning environment as supportive and engaging. On the metalevel, the optimal learner continuously monitors every aspect of the learning process to achieve their learning goals (i.e., monitoring) and makes necessary adjustments to the learning process as needed (i.e., control) [19].

### Stress

*Stress* is characterized by an individual’s physiological and psychological reactions to a stressor. It manifests as a response to perceived demands or threats, disrupting the person’s internal equilibrium. This response can be observed through various physiological, behavioral, emotional, and cognitive responses or reactions in the individual [29]. Lazarus and Folkman’s Transactional Stress Model [30, 31] provides a comprehensive framework for understanding stress as a dynamic process involving ongoing transactions between an individual and their environment. The model emphasizes the role of cognitive appraisal during the stress response: primary appraisal involves evaluating whether an event is a threat, while secondary appraisal considers available coping resources and options to deal with the stressor. This appraisal process then leads to coping responses. In a clinical setting, for example, when a student encounters a new clinical task for the first time, they might initially perceive the situation as a threat due to the perceived demands of the task (primary appraisal). During the subsequent secondary appraisal, the student assesses their available resources, such as confidence in their abilities or previous experience with similar tasks, to evaluate how to approach the situation and determine whether their resources are adequate to meet its demands.

Regarding our operationalization of stress, while stress can be evaluated using biological markers or subjective measures, such as questionnaires and visual analogue scales, this study focuses on the psychological aspect by using self-reports to assess students’ subjective experiences of stress.

### Academic stress in higher education

*Academic stress* is a critical issue that affects a significant portion of the higher education student population [32]. Academic stress in higher education is characterized by stress responses that students experience due to the high demands and expectations prevalent in tertiary education settings. These demands often include challenging coursework, rigorous academic standards, and looming deadlines, which can lead to heightened levels of stress [33]. This form of stress is associated not only with poor mental and physical health [32, 34, 35] and low academic performance [1, 36, 37] but also with overall poorer quality of life and well-being [32, 38]. The pressures associated with higher education can also have a detrimental effect on students’ motivation and engagement, potentially leading to burnout, a state characterized by emotional exhaustion, cynicism, and feelings of reduced accomplishment [39, 40]. Furthermore, stress can impair cognitive functions such as concentration and memory [41, 42], which could be especially crucial in higher education settings, which constantly demand the use of higher cognition from students, further hindering academic achievement [1].

Undergraduate health science students face additional stressors when transitioning to clinical or workplace settings, including the practical application of knowledge and development of practical clinical skills [5, 43], the navigation of healthcare information systems [44], the management of emotional demands [45–47], and interaction with patients, while also working to develop professional autonomy [43]. Exposure to patient suffering and ethical dilemmas can lead to emotional exhaustion and increased stress, which affects well-being as well as learning outcomes [48]. Furthermore, balancing academic and workplace demands may result in stress related to time management and burnout [39, 40]. Overall, the transition to clinical learning presents significant challenges for health science undergraduates [49].

### Interplay of SRL and stress

A theoretical rationale to explain the influence of stress on learning processes is through the means of CLT [6]. According to CLT, the human brain has a limited capacity for processing information. When students experience high levels of stress, their cognitive resources might be diverted toward managing their emotional state, which increases extraneous cognitive load [7, 50] and potentially reduces the resources available for learning-related tasks, such as setting goals, monitoring progress, and employing and adjusting strategies. Boekaerts [51, 52] highlights that under significant stress, students may shift their focus from academic tasks to emotional coping mechanisms, further exacerbating the cognitive burden on them. As a result, effective SRL is impaired, leading to poorer learning outcomes and potentially creating a cycle of increased stress and reduced academic performance. On the other hand, SRL may mitigate against academic stress by improving academic outcomes. Research indicates that students who actively employ SRL strategies are more likely to achieve their academic goals [2, 8], which, in turn, could serve as a protective factor against academic stress [4]. Moreover, effective SRL helps students manage their workload more efficiently, reducing the likelihood of last-minute cramming or falling behind, both of which are common sources of academic stress [32]. By breaking down tasks, planning ahead, and regularly assessing their understanding, students can maintain a steady and manageable pace in their studies, which helps prevent the accumulation of stress. Previous studies have shown that time management strategies play a significant role in reducing stress [9]. Previous research investigating the link between stress and SRL in workplace and higher education settings, mainly cross-sectional, has correlated various aspects of SRL and perceived stress (e.g [53–56]), or explored unidirectional effects (e.g [57]). However, there is a lack of research that integrates and explores the bidirectional relationships of the multifaceted areas of SRL with stress in higher education and workplace environments in one comprehensive study. Although SRL and stress seem to be linked to academic achievement [1, 2], it is not entirely clear whether and how they influence each other reciprocally. In the following section, we will discuss the relevant existing literature for each SRL area.

#### Cognitive learning strategies

Stress is known to adversely affect cognitive processes through its neurological impact on the brain [41, 42]. This effect could be particularly crucial in educational environments, such as higher education and workplace settings, where higher cognition is constantly demanded from students. The frequent use of strategies involving self-regulation in undergraduates is associated with lower levels of academic stress [58]. Additionally, Lakkonen and Nevgi [59] observed that third-year students who experience higher levels of academic stress tend to engage more in reflective learning. Interestingly, this correlation was not observed among first-year students, suggesting that the relationship between academic stress and reflective learning may develop or become more apparent later in the academic journey. Li et al. [60] reported that stress significantly and directly impacts both task and contextual performance. Additionally, their research highlights a notable indirect effect of stress on these types of performance through the mechanism of cognitive SRL strategies. Broks and colleagues [61] used latent profile analysis to examine links between test anxiety, SRL, and stress. Their results showed that students with low test anxiety and high SRL abilities experienced lower stress levels than did those with high test anxiety and high SRL abilities and those with moderate test anxiety and low SRL abilities. Regarding the directional effect of cognitive SRL strategies on academic stress, Häfner, Stock, and Oberst [9] highlight the benefits of time management strategies in reducing perceived stress.

#### Motivation

Previous research conducted in higher education and workplace settings has shown that stress negatively impacts undergraduates’ motivation and engagement [53–57, 62]. This reduction in motivation and engagement complicates students’ learning experiences, potentially resulting in less effective learning and subsequently hindered academic success [40, 57]. Conversely, past research has suggested that students who demonstrate high levels of self-efficacy, a core element of students’ motivation, experience lower levels of perceived stress [63], indicating a potential bidirectional relationship.

#### Emotion

In terms of emotions, negative emotions such as anxiety and anger are closely intertwined with stress at the neurological level. While anxiety is sometimes seen as a reaction to stress, stress can trigger a range of physiological, psychological, and emotional responses [29]. Consequently, in the present study, we conceptualize anxiety as a component of negative emotion and, in turn, as part of SRL, without further addressing its overlap with stress in more detail but refer the interested reader to Daviu et al. [64] for more information about this issue. In this context, negative emotions could influence students’ stress levels and academic performance by activating key brain regions, notably the amygdala and the prefrontal cortex [64]. Studies investigating the well-being of undergraduate medical students often investigate anxiety and stress in conjunction, both of which are consistently listed as factors that appear to be heightened in such populations [34, 65] and seem to increase during education [65–68], especially at the end of the preclinical phase [65]. The current medical education literature suggests a bidirectional relationship between stress and anxiety. Studies indicate that undergraduates with higher anxiety levels tend to experience increased stress [65]; conversely, those with elevated stress levels often report greater anxiety [63, 65]. Positive emotions also play an important role in regulating the stress response, specifically by restoring resources and enhancing coping mechanisms [69]. In that context, higher levels of situational stress in undergraduates have been previously linked to increased negative emotion and reduced positive emotion [58, 70], while suppressing the expression of positive emotion in nurses was previously found to be associated with greater subjective stress [71].

#### Students’ perception of the learning environment

The major role of the context in which undergraduates work and learn has been increasingly recognized in the field [19, 26–28]. Past studies have indicated that undergraduates’ perceptions of a supportive and well-structured learning environment not only lead to more favorable academic achievement [72] but also play a critical role in mediating stress and burnout. For instance, Sum et al. [73] reported that such positive perceptions of the learning environment seem to mediate the relationship between perceived stress and burnout. Conversely, Meriläinen and Kuittinen [74] observed that negative perceptions of the learning environment correlate with increased levels of burnout, underscoring the importance of a positive educational setting. Despite these findings, there remains a noticeable research gap regarding directional effects between undergraduates’ perceptions of the learning environment and stress.

#### Metalevel

The regulation of the aforementioned SRL-areas [19, 25] can significantly impact students’ stress levels and overall academic experience. Efficient cognitive strategy management can decrease cognitive overload and reduce stress [75], and the use of metacognitive SRL strategies has been previously shown to negatively affect academic stress [76]. Conversely, maladaptive metacognition has been shown to positively correlate with perceived stress [77].

Furthermore, improved regulation of motivation not only enhances academic performance [78] but could also lead to lower stress levels in students. Grunschel and colleagues [79] reported that motivational regulation can decrease academic procrastination, a significant source of academic stress, potentially contributing to stress reduction [80]. However, certain specific maladaptive avoidant motivational regulation strategies might have the opposite effect by increasing stress and negatively impacting academic performance [79].

In the realm of emotion regulation, cognitive reappraisal to increase positive emotion has been previously found to be negatively associated with stress [71]. Moreover, emotional coping, akin to emotional regulation in the SRL framework, plays a crucial role in managing stress and involves strategies to handle emotional responses. This concept is a key element of Lazarus and Folkman’s transactional stress model and emphasizes how individuals actively manage and respond to stressful situations [30, 31]. The significant effects of emotional coping on stress have been documented in numerous studies (meta-analyses: [81, 82]). While emotion regulation refers to a broader array of strategies to manage one’s own emotions, not necessarily in response to stressors, the overlap of both constructs makes it reasonable to considering literature involving coping when examining the relationship between emotion regulation and stress [83, 84].

Regarding the regulation of the perception of the learning environment, actively engaging with and positively influencing the learning environment could create a more supportive context, potentially further mitigating stress. However, empirical studies investigating this topic have not been reported in the literature.

## Goals

There is a lack of previous comprehensive studies investigating the bidirectional relationships between aspects of undergraduates’ workplace SRL and stress. Building on this gap, the present study is designed to examine the nature and directionality of the longitudinal relationship between stress and various aspects of SRL in undergraduate students in a workplace environment over a 15-week period, with a particular focus on pairwise measurement points (i.e., week-to-week variations).

We present the following hypotheses for each SRL-aspect:

1) Stress in the previous week will impact the SRL-aspect in the current week, taking the SRL-aspect measurement in the prior week into account.

2) The SRL-aspect in the previous week will impact stress in the current week, taking the stress measurement in the prior week into account.

We refrain from specifying directional hypotheses due to the limited differentiation in the literature compared to our comprehensive SRL assessment. However, accounting for the general trend in previous studies, which predominantly indicated negative associations between stress and various aspects of SRL, we expected negative associations between stress and most SRL-aspects.

## Method

### Participants

The results should accurately reflect a diverse population of health science students regarding their cognitive and metacognitive learning strategies, motivation, emotions, perception of the learning environment and stress. Therefore, instead of distributing questionnaires to various institutions, which might have resulted in a possibly biased sample primarily composed of motivated high achievers, we focused our efforts on reaching a substantial portion of a relevant student cohort within a single institution.

All 192 veterinary students at Vetmeduni Vienna enrolled in the ‘Clinical Rotation I’ course (representing their workplace placements) during the data collection period participated in the study. Three students did not consent to their data being used for research purposes, and one student was excluded from further analysis because of a high proportion of missing values (> 50%), resulting in a final sample size of *N* = 188 (83.5% female, 16.5% male, 0% non-binary; age range: 21–39 years, *M* = 24.60, *SD* = 2.92).

### Measures

#### SRL-aspects

To evaluate undergraduates’ experiences of workplace learning, we utilized single-items derived from the Workplace Learning Inventory (WLI; [19]), except for emotion, where we used the scales. The WLI includes a range of scales for assessing workplace learning in the areas of cognition, motivation, and emotion (the items measuring emotion in the WLI were adapted from Duffy et al. [85] and subsequently combined into new scales in the WLI; see [19]), perceptions of the learning environment (i.e., context), and monitoring and regulation of the aforementioned areas on a metalevel (i.e., monitoring and control). The psychometric properties of the single-items were previously tested using an independent sample [86]. Reliability of all items was adequate [87, 88]; however, validity presented a mixed picture. While the relationship within the nomological network was satisfactory across all items, the information reproduction for most items was found to be lacking [89]. Consequently, Steinberg et al. [86] suggest that when interpreting results obtained from using these items, rather the specific wording of the respective single-items should be considered than the broader definitions of the aspects as outlined by the WLI. See Table 1 for an overview of administered single-items, scales and their reliability.

**Table 1:** Administered single-items/scales from the workplace learning inventory: reliability and relationship within the nomological network

Area/sub-area/aspect	Single-item	Reliability ω^2^	Relationship within the nomological network
Cognition			
Cognitive learning strategies			
Preparation	Before I came to the workplace, I worked to acquaint myself with relevant topics.	0.745	acceptable
Attention	At the workplace, I stayed concentrated while completing practical medical tasks.	0.731	acceptable
Rehearsal	At the workplace, I consciously committed important information to memory.	0.694	acceptable
Elaboration	At the workplace, I tried to connect the practical medical tasks to what I had previously learned.	0.828	acceptable
Clarification	At the workplace, I asked for advice when something was unclear.	0.841	acceptable
Consolidation	After leaving the workplace (no matter if, e.g., 10 min–2 h afterward), I further deepened what I had learned and practiced.	0.666	acceptable
Proximal metacognitive learning strategies			
Planning	Before I came to the workplace, I thought about what medical cases I could expect.	0.706	acceptable
Reviewing	At the workplace, I recapitulated what I had practiced or learned in order to determine whether everything is clear to me.	0.588	acceptable
Reflection	After leaving the workplace (no matter if, e.g., 10 min–2 h afterward), I reflected on what I would do differently next time.	0.755	acceptable
Motivation			
Expectancy of success	I am confident that this week I will be able to do what is asked of me.	0.745	acceptable
Situational interest	This week I found the tasks interesting.	0.769	acceptable
Mastery goal approach	This week it was important to me to expand my knowledge.	0.771	acceptable
Performance goal approach	This week it was important to me to practice exactly what the instructors are looking for when evaluating my performance.	0.778	acceptable
Effort	This week I made an effort.	0.757	acceptable
Attention control	This week I was not focused while practicing and studying.	0.740	acceptable
Proactive attitude	This week I took advantage of opportunities to gain hands-on practice.	0.832	acceptable
Emotion (*Item stem for items regarding emotion*: Think about your learning and practicing this week: To what extent were you …)			
Negative Emotions (scale)	… anxious, frustrated, angry, sad?	0.792	acceptable
Positive Emotions (scale)	… proud, happy, hopeful, curious?	0.788	acceptable
Context			
Organizational framework conditions	I had the impression that the clinic/facility was well-organized, so that students encountered good contextual conditions.	0.814	acceptable
Supervisory quality	The instructors offered me opportunities to further develop.	0.694	acceptable
Staff support	I was supported by members of the staff working here.	0.724	acceptable
Cognition metalevel			
Monitoring	This week I paid attention to whether my study and practice behavior would help me reach my goal.	0.757	acceptable
Control	This week I changed the way I study or practice when I noticed that I was not improving.	0.638	acceptable
Motivation metalevel			
Monitoring	This week I paid attention to how motivated I am.	0.724	acceptable
Control	This week I changed something when I noticed that I was not motivated.	0.659	acceptable
Emotion metalevel			
Monitoring	This week I reflected on my feelings while studying and practicing.	0.876	acceptable
Control	This week I changed something when I noticed that my feelings (e.g., fear or anger) were impeding me while studying or practicing.	0.687	acceptable
Context metalevel			
Monitoring	This week I reflected on what contextual conditions^a^ accompany my studying and practicing.	0.834	acceptable
Control	This week I changed how I study or practice in order to better adapt to contextual conditions^a^. ^a^(organizational conditions, instructors, other students, on-site staff, equity concerns)	0.658	acceptable

*Note* All items were administered using five-point Likert scales: 1 = *does not apply at all*, 2 = *does not apply*, 3 = *partly applies*, 4 = *applies*, 5 = *fully applies*; Emotion: 1 = *not at all*; 2 = *a little*; 3 = *moderately*; 4 = *fairly*; 5 = *very much.* Single-items are a back-and-forth translation from the original items in the German language. Cognition area items were administered daily (and later aggregated for analysis), while all other measures were administered weekly. Reliabilities and relationships within nomological networks were based on Steinberg et al., 2023

This study was part of a larger project on SRL in the workplace, including a diary study. Thus, to mitigate survey fatigue and avoid overburdening participants, only cognition items were assessed on a daily basis. All the other individual items and the full scales were assessed on a weekly basis at the end of the week, except for ‘expectancy of success’, which was gauged at the beginning of each week.

Daily data were aggregated into weekly data for analysis. Some single-items were combined into scales in our analysis based on both theoretical and statistical considerations. First, the individual items for context, *organizational framework conditions*,* supervisory quality*, and *staff support*, were merged into a single factor. We excluded *peer support* and *equal treatment* because of limited variance and theoretical reasons (notably, more than 80% of our participants were female, and peer support was anticipated to remain stable since the students worked in consistent groups throughout the course). Second, at the metalevel and drawing from the work of Kim and colleagues [90], who found shared variance among the regulation of cognition, motivation and emotion, as well as that of Wirth et al. [25], who consider monitoring and control to be central at the metalevel, we combined all monitoring items into one factor and all control items into another factor. These groupings were subsequently validated through multilevel confirmatory factor analysis (refer to the Results section for more details).

#### Stress

To assess students’ stress during their workplace placements, we used a single-item measure: ‘Please reflect on this week: How stressed did you feel this week during Clinical Rotation I?’. Participants responded on a 5-point Likert scale (1 = *not at all*; 2 = *a little;* 3 = *moderately*; 4 = *substantially*; 5 = *very much*). This item was administered weekly at the end of each week.

### Procedure

The present study employed a longitudinal diary design over the course of an entire semester and was conducted at the University of Veterinary Medicine Vienna (Vetmeduni Vienna). Workplace placements, a key component of the institution’s program, are usually completed by students during their ninth semester, and emphasize integrating students into clinical practice. For most students, these placements are their first prolonged exposure to a clinical work environment, representing a significant shift from primarily learning in a classroom environment to a more practical, clinical focus. During this phase, students apply their knowledge in real-world clinical settings, developing practical abilities and skills needed for their future profession. The students were organized into groups of eight and systematically rotated weekly among 15 distinct work placements, which were characterized by distinct clinical and teaching staff as well as subject areas. Details about these placements can be found in the Appendix (Additional File 1). Students were briefed about the study’s objectives and the intended use of its findings through an informational event before data collection. Data collection was conducted from the 25th of July 2022 to the 27th of January 2023 using the online survey tool Unipark© (Unipark EFS Survey, Globalpark, Cologne, Germany). Participants were provided with a daily link, which allowed them to access the survey through a web browser on their preferred device. Participants who did not complete the survey were reminded on the same day. Additionally, access to the questionnaire was restricted to the current day’s survey only. Reflecting on their learning and stress experiences by filling in the survey or completing an alternative task was implemented into the curriculum of the course and supported the learning goal ‘reflecting on one’s own learning and practicing’. Written Informed consent was obtained from the students for their participation in the study and for the use of their data.

To foster complete and high-quality data, several measures were taken. First, students and teachers were informed about the relevance of this topic. Second, students were given time at the workplace to complete the diary. Third, student contact persons ensured good communication between the students and the project team in the case of technical or motivational problems. Fourth, at the end of the course, students were provided with a personalized report on their learning and stress experiences during the semester, including practical recommendations for further improvement. Finally, in recognition of their participation, students were invited to a social event and awarded a voucher.

### Data analysis

We employed multilevel vector autoregressive (M-VAR) models [91] to explore the dynamic, bidirectional relationships between stress and various aspects of SRL in workplace settings. While M-VAR models yield estimates for both autoregressive effects, which assess the (rank-order) stability of each variable over time, and cross-lagged effects, which investigate the directional influences between stress and SRL-aspects, the particular focus of the present study was on cross-lagged effects. The data were gathered over 15 weeks, with the points of interest being pairwise measurement points (i.e., week-to-week variations). To ensure the validity of the variable categorizations, we utilized multilevel confirmatory factor analysis (MCFA). To evaluate the proportion of variance attributed to differences between and within individuals, Intraclass Correlation Coefficients (ICCs) were calculated for each SRL-aspect and stress level. All analyses were performed with Mplus Version 8.7 [92] using the robust maximum likelihood estimation method. A significance level of *p* < .05 was used to determine the statistical significance of the findings. Concerning the interpretation of cross-lagged path estimates, we referred to the guidelines proposed by Orth et al. [93], which suggest benchmark values of 0.03 (small effect), 0.07 (medium effect), and 0.12 (large effect).

### Missing data

In our dataset, 4.66% of the data were missing. The proportion of missing data varied across the 36 variables in our study, ranging from 1.22 to 7.78%. To address this, we employed the full information maximum likelihood (FIML) method under the assumption that the data were missing at random (MAR), as recommended by Enders [94].

## Results

### Preliminary analyses: ICC and multilevel confirmatory factor analyses

To evaluate the proportion of variance attributed to differences between and within individuals, ICCs were calculated for each SRL-aspect and stress level. The ICCs for the SRL-aspects ranged from 0.02 to 0.88, indicating that a small to large proportion of the variance was due to between-person differences, with notable variability across different SRL-aspects. For stress, the ICC was higher (0.25), suggesting that a significant amount of the variance in stress levels was attributable to between-person differences (for more details, see Additional file 2).

Next, we conducted MCFA for ‘context’ and a proposed shared metalevel [19, 90], encompassing monitoring and controlling of the cognition, motivation, emotion, and context areas. Table 2 contains model fits, reliability and ICC ranges of the two models. The model fits ranged from ‘acceptable’ to ‘good’ or ‘very good’ according to common cutoff criteria [95].

**Table 2:** MCFAs for context and metalevel: model fits, reliability and ICC range

	χ2	df	CFI	TLI	RMSEA	SRMR		ICC Range
Model						Within	Between	
Context^a^	72.65	9	0.97	0.96	0.05	0.02	0.00	0.143–0.319
Meta-Level^b^	229.75	25	0.91	0.90	0.06	0.04	0.00	0.384–0.482

*Note N* = 5250 total observations from 188 students

^a^2-factor model: Context (single-items: Organizational Framework Conditions, Supervisory Quality and Staff Support; ω = 0.856) and another latent context factor (single-items: Peer Support and Equal Treatment) excluded from further analysis due to lack of variance and theoretical considerations. *N* = 2624 observations

^b^2-factor model: Monitoring (single-items: Cognition Monitoring, Motivation Monitoring, Emotion Monitoring, Context Monitoring; ω = 0.624) and Control (single-items: Cognition Control, Motivation Control, Emotion Control, Context Control; ω = 0.725). Two residual covariances were specified due to similarities in item means. *N* = 2626 observations

### Relationship between aspects of SRL and stress

Multilevel vector autoregressive (M-VAR) models were constructed for each SRL-aspect individually. The results from the M-VAR models are depicted in Table 3 and summarized below.

**Table 3:** Results of the multilevel vector autoregressive models: estimates, standardized estimates, standard errors and R²

SRL-Area	SRL- Aspect	Cross-lagged effect						Autoregressive effect						*R*²		*N*
		SRL-aspect → stress			Stress → SRL-aspect			Stress			SRL-aspect			SRL-aspect	Stress	
		Est.	SE	Std. Est.	Est.	SE	Std. Est.	Est.	SE	Std. Est.	Est.	SE	Std. Est.	Est.	Est.	
Cognition	Preparation	**-0.10**	0.03	**-0.09**	-0.03	0.02	-0.03	**0.14**	0.03	**0.14**	0.01	0.02	0.01	0.00	0.03	2763
	Attention	-0.03	0.04	-0.02	**-0.04**	0.02	**-0.07**	**0.14**	0.03	**0.14**	**0.10**	0.03	0.09	0.01	0.02	2763
	Rehearsal	**-0.07**	0.04	**-0.05**	-0.02	0.02	-0.03	**0.14**	0.03	**0.13**	**0.07**	0.03	**0.07**	0.01	0.02	2763
	Elaboration	-0.03	0.04	-0.02	**-0.05**	0.02	-0.07	**0.14**	0.03	**0.14**	**0.09**	0.03	**0.08**	0.01	0.02	2763
	Clarification	-0.06	0.04	-0.04	**-0.04**	0.02	**-0.05**	**0.14**	0.03	**0.13**	**0.09**	0.03	**0.09**	0.01	0.02	2763
	Consolidation	-0.02	0.02	-0.02	**-0.05**	0.02	**-0.05**	**0.14**	0.03	**0.14**	**0.09**	0.03	**0.09**	0.01	0.02	2764
	Planning	**-0.13**	0.03	**-0.11**	-0.01	0.02	-0.01	**0.15**	0.03	**0.14**	**0.11**	0.03	**0.11**	0.01	0.03	2763
	Control	-0.06	0.03	-0.04	**-0.04**	0.04	**-0.05**	**0.14**	0.03	**0.13**	**0.12**	0.03	**0.12**	0.02	0.02	2763
	Reflection	**-0.06**	0.02	**-0.05**	-0.04	0.02	-0.04	**0.15**	0.03	**0.15**	**0.14**	0.03	**0.13**	0.02	0.02	2764
Motivation	Expectancy of Success	**0.10**	0.03	**0.09**	-0.02	0.03	-0.02	**0.18**	0.03	**0.17**	**-0.06**	0.03	**-0.06**	0.00	0.03	2758
	Situational Interest	-0.01	0.02	-0.01	**-0.09**	0.03	**-0.08**	**0.14**	0.03	**0.14**	0.05	0.03	0.05	0.01	0.02	2727
	Mastery Approach	-0.05	0.03	-0.04	-0.03	0.02	-0.03	**0.14**	0.03	**0.13**	**0.14**	0.03	**0.13**	0.02	0.02	2727
	Performance Approach	**-0.09**	0.02	**-0.08**	-0.02	0.02	-0.02	**0.14**	0.03	**0.14**	**0.08**	0.03	**0.08**	0.01	0.03	2727
	Effort	-0.05	0.03	-0.04	**-0.05**	0.02	**-0.06**	**0.14**	0.03	**0.14**	0.05	0.03	0.05	0.01	0.02	2727
	Attention Control	0.03	0.02	0.03	0.05	0.03	0.05	**0.14**	0.03	**0.14**	-0.03	0.03	-0.03	0.00	0.02	2727
	Proactive Attitude	-0.05	0.03	-0.04	**-0.07**	0.02	**-0.08**	**0.14**	0.03	**0.13**	**0.10**	0.03	**0.09**	0.02	0.02	2727
Emotion	Scale^a^: Positive Emotion	**-0.08**	0.04	**-0.06**	-0.02	0.02	-0.03	**0.13**	0.03	**0.12**	**0.12**	0.03	**0.12**	0.02	0.02	2728
	Scale^b^: Negative Emotion	0.02	0.04	0.01	**0.09**	0.02	**0.12**	**0.14**	0.03	**0.13**	**0.06**	0.03	**0.06**	0.03	0.02	2728
Context	Scale^c^	-0.03	0.03	-0.03	**-0.11**	0.02	**-0.11**	**0.13**	0.03	0.13	0.06	0.03	0.06	0.02	0.02	2726
Monitoring	Scale^d^	-0.04	0.03	-0.03	0.02	0.02	0.02	**0.14**	0.03	**0.14**	**0.20**	0.03	**0.19**	0.04	0.02	2727
Control	Scale^e^	-0.05	0.03	-0.05	0.03	0.02	0.03	**0.15**	0.03	**0.15**	**0.14**	0.03	**0.13**	0.02	0.02	2727

*Note N* = 57,625 total observations from 188 students; Est. = Unstandardized estimate; SE = Standard error; Std. Est. = Standardized estimate; Statistically significant results at α = 0.05 are shown boldface. ^a^The scale includes the emotions: pride, happiness, hope and curiosity. ^b^The scale includes the emotions anxiety, frustration, anger and sadness. ^c^The scale includes the aspects organizational framework conditions, supervisory quality and staff support. ^d^The scale includes monitoring of the cognition-, motivation-, emotion- and context area. ^e^The scale includes the controlling of the cognition-, motivation-, emotion- and context area

R² indicated that SRL-aspects explained between 0.00 and 0.04% of the variance in Stress and Stress explained between 0.02 and 0.03% of the variance in SRL-aspects. Ten of the 21 path estimates for the impact of stress from the prior week on individual SRL-aspects of the current week were statistically significant, and 7 out of 21 path estimates for the impact of the SRL-aspects from the prior week on stress of the current week were statistically significant. Previous stress negatively impacted SRL (except for ‘negative emotion’, for which prior stress had a positive effect), and the SRL-aspects from the previous week had a negative impact on stress (except for ‘expectancy of success’, for which prior SRL-aspect levels had a positive effect).

The cross-lagged effect estimates for SRL-aspects impacting stress were predominantly medium-sized, with ‘planning’ exhibiting a large effect (ranging from 0.07 to 0.13, *M* = 0.09). Conversely, the effects of stress on individual SRL-aspects were mostly small (ranging from 0.04 to 0.11, *M* = 0.06), except for ‘situational interest’ (-0.09), ‘negative emotion’ (0.09), and ‘context’ (-0.11), which exhibited a medium effect.

Regarding autoregressive effects, only one SRL-aspect, ‘expectancy of success’, showed a negative and significant estimate, while ‘attention control’ also showed a negative estimate but was not statistically significant. According to Schuurman et al. [96], the negative autoregressive effect indicates that if “expectancy of success” is higher at one measurement occasion, it is likely to be low at the next. All other autoregressive estimates of the other models were positive.

## Discussion

The aim of the present study was to examine the relationships between various aspects of SRL and the stress of undergraduate health science students in the workplace. We used M-VAR models, with a particular focus on the weekly development of these relationships, i.e., how stress from the previous week impacts aspects of SRL in the current week and how SRL-aspects from the previous week impact stress in the current week. In the following, to maintain focus and brevity, we will not cover every specific result in detail. Instead, we will try to offer a broader perspective by discussing key examples that illustrate the primary findings.

The results showed that some of the SRL-aspects impact stress, while others were impacted by stress. There was no SRL-aspect with a reciprocal relationship with stress (i.e., no SRL-aspect both affected stress and was affected by stress). The directions of cross-lagged effects largely conformed to the expected patterns of mainly negative cross-lagged paths. Furthermore, SRL-aspects had a stronger impact on stress than previous stress had on individual SRL-aspects. The findings of the present study underscore the necessity of a detailed, nuanced approach to studying SRL in workplace environments, as certain aspects of the same SRL areas appear to impact stress, while others are impacted by stress. The results provide a largely uniform picture of the direction of the association: the greater the stress in the previous week was, the worse the learning in the current week was; conversely, the better the learning in the previous week was, the lower the stress in the current week was. These findings align with the current literature in the field [53–57, 62, 63, 65]. They also align with the general assumptions of cognitive load theory (CLT; [6]), which posits that an individual’s cognitive capacity is limited and can be overwhelmed by excessive demands. According to CLT, lingering stress from one week can increase cognitive load, thereby impairing the ability to effectively engage in self-regulated learning strategies in the subsequent week [7, 50]. Exceptions to this large pattern of negative links were negative emotions (which was anticipated; see, e.g., Moutinho et al. [65]) and expectancy of success. The latter was particularly unexpected, as a high level of expectancy of success or self-efficacy regarding a task is typically linked to resilience and task-oriented coping [97] and more successful task completion, leading to reduced stress [98]. One possible explanation for this unexpected result might be that students overestimated their abilities, leading to a disparity between their expectations and the actual demands of the task, which is a known phenomenon [99, 100]; however, it seems unlikely that this effect would occur universally among all students. Furthermore, aside from self-efficacy, all other motivational aspects showed a negative link with stress. Specifically, performance approach motivation negatively influenced stress in the following week, while stress from the previous week had a detrimental effect on situational interest, effort, and proactive attitude in the current week. This time-lagged effect suggests that stress does not merely have immediate consequences [57], but can also disrupt motivation over an extended period. High levels of stress can tax cognitive and emotional resources [6, 7], making it more difficult for students to maintain intrinsic motivation and engagement in their activities. The results further showed that students currently motivated by performance approach goals—striving to demonstrate competence by outperforming their peers [101]—experience reduced future stress. Successfully achieving these goals might provide them with a sense of accomplishment and validation [102], which could help to buffer against current and future stress. In contrast, mastery approach goals, which focus on personal growth and skill development, did not exhibit a significant cross-lagged interaction with stress. This may be because mastery goals emphasize long-term self-improvement rather than immediate external validation [101]. As a result, they may not provide the quick stress relief that can be observed in short-term, week-to-week variations.

A more nuanced picture emerges with a differentiated view of the individual SRL-aspects: the results show that SRL-aspects that significantly influenced stress differed from those significantly influenced by stress. A possible framework for understanding our findings is offered by aligning them with the foundational concepts of SRL established by Zimmerman [13], which divides the learning process into forethought, performance, and self-reflection phases. Aspects more integral to undergraduates’ workplace experience (performance phase), such as attention, control, clarification, situational interest, effort, proactive attitude, perception of the learning environment, and negative emotion, seem more susceptible to being negatively influenced by stress of the previous week, as indicated by the significant path coefficients in our analyses. This effect may result from students’ direct exposure to the distinct and challenging stressors inherent in clinical environments during the current week, which in turn impacts their stress levels [4, 5, 103]. These clinical stressors could deplete students’ mental and emotional resources and compromise their capacity to cope with residual stress from the previous week. In contrast, aspects that are predominantly relevant before or after being in the workplace (forethought and self-reflection phase), such as preparation, planning, and reflection, appeared to significantly diminish future stress levels. In the forethought phase, students engage in anticipatory strategies such as goal setting and planning, which could foster resilience and better stress management in upcoming tasks or challenges. This proactive approach allows students to mentally prepare for potential stressors, potentially mitigating their impact in the next week [104]. Similarly, during the self-reflection phase, students analyze their past performance and learning experiences. This reflective practice enables them to identify stressors and their associated coping mechanisms [105], leading to improved stress management strategies in future scenarios.

Regarding effect sizes, the present results suggest differences in effect sizes between the impact of individual SRL-aspects on stress and the reverse. Specifically, significant cross-lagged paths from SRL-aspects to stress were predominantly of medium size, with ‘planning’ exhibiting a large effect. Conversely, the effect sizes for the influence of stress on individual SRL-aspects were small to medium. Medium effect sizes were specifically observed for ‘situational interest’, ‘negative emotion’, and ‘context’ (i.e., perception of the learning environment). This could indicate that stress leads to a diminished sense of involvement and curiosity in academic tasks, reflecting a lagged impact on undergraduates’ immediate academic interests, possibly due to the time they need to process and evaluate their stress experiences. Similarly, the stronger link with negative emotion is not surprising considering the shared neural pathways between stress processing and emotional states [64]. Given that stress is known to accumulate over time, it is not surprising that stress experienced in the previous week continues to affect students’ negative emotional states in the following week. In comparison, the present results also indicate the complementary buffering effect of positive emotions on stress, as noted in the literature [71]. This effect is widely recognized and can be explained by the role that positive emotions play in restoring resources and enhancing coping mechanisms [69]. Despite various studies indicating a link between the regulation of various SRL-areas and stress [75, 76, 79, 80], we found no cross-lagged effects for the control or monitoring scales in our study.

### Strengths

Our study’s strengths lie in its dataset, which features longitudinal data collected over 15 weeks in diverse workplace settings. This extensive data collection enables us to capture week-to-week variations effectively. Additionally, our comprehensive approach, encompassing 21 aspects of SRL, provides a broad and detailed perspective. The utilization of single-items, derived from the Workplace Learning Inventory (WLI; [19]), a newly developed tool specifically designed for assessing SRL in workplace environments, further enhances the validity and relevance of our findings. All single-item measures were previously tested for reliability and validity in an independent sample [86].

### Limitations and future directions

Despite the strengths mentioned above, this study has certain limitations. The study was conducted at a single institution, which may constrain the wider applicability of our findings. While this approach minimizes the risk of a biased sample, which could occur if surveying multiple institutions (potentially attracting mostly highly motivated high achievers with positive attitudes towards their learning), future research should explore conducting studies across multiple institutions to achieve a broader and more generalizable understanding of the phenomena. A significant limitation is our reliance on single-item measures for certain constructs. While this approach can help maintain participant engagement and make longitudinal investigations feasible without overburdening respondents, these measures have inherent drawbacks [106]. Although single-item measures are less ambiguous and more straightforward for participants, they do not allow for the estimation of internal consistency, raising concerns about potentially low reliability. Furthermore, they may not adequately capture complex psychological constructs or provide fine-grained distinctions between individuals as do multi-item scales. Consequently, this methodological approach might limit the depth and detail of our insights into some of the individual aspects of SRL.

In contrast, for emotions, we used multi-item scales but did not differentiate between individual emotions. Instead, we aggregated negative and positive emotions into scales to provide a broad understanding of their relationships with stress. Future research could benefit from exploring individual emotions to better understand their distinct interactions with stress. For example, while we conceptualized anxiety as a component of negative emotions within SRL, examining anxiety separately could provide valuable insights into the interplay of these constructs. Although there is significant literature on the overlap between stress and anxiety (e.g [107–109]). exploring these differences in detail was beyond the scope of this study. Additionally, there were no statistically significant metalevel cross-lagged effects in our study, contrasting with previous findings in the literature [75, 76, 79, 80]. This could be a consequence of our methodology: all monitoring aspects of the individual areas were combined into one scale and all the control aspects were combined into another scale. While this approach enhances reliability, a separate analysis of each area of SRL might uncover more subtle relationships.

Moreover, while our study measured students’ perceptions of the learning environment, we did not explicitly evaluate it by the means of external regulation and future studies might benefit from considering the role of external regulation alongside self-regulation to better understand students’ learning processes [110, 111]. Assessing both, self-regulation and external regulation processes, might be particularly relevant in healthcare education, where both personal and contextual factors are crucial in assessing and improving self-regulatory behaviors [111]. Furthermore, it would be interesting to see how external regulation affects stress and its relationship with self-regulation.

Lastly, while the focus of our study was on weekly variations, different temporal foci should be considered by future studies. Daily or within-day measurements could offer valuable insights into short-term fluctuations and more immediate changes in the relationship between SRL and stress. This may help capture dynamic patterns that are not visible at a weekly level, providing a deeper understanding of how stress and SRL interact over shorter timeframes.

### Scientific implications

The interaction between SRL-aspects and stress over time appears to vary depending on the SRL phase [13] to which they are more integral. This finding is significant because the forethought and self-reflection phases are often overlooked in workplace learning, particularly in medical education. Traditionally, the focus has been primarily on learning that occurs directly in the workplace environment [27, 28], with little consideration given to the preparation and reflection that occur before and after the workplace activities. However, our findings suggest that SRL-aspects associated with the performance phase—such as attention, control, clarification, situational interest, effort, proactive attitude, perception of the learning environment, and negative emotion—are more susceptible to the negative effects of stress from the previous week (see the beginning of the discussion for an explanation why this might be the case). In contrast, SRL-aspects related to the forethought and self-reflection phases, such as preparation, planning, and reflection, are shown to significantly reduce future stress levels. This highlights the importance of considering these phases when aiming to prevent stress or enhance SRL competencies.

### Practical implications

The inclusion of SRL skill development in educational programs is crucial for equipping students with the necessary tools to effectively navigate workplace challenges, and the development of those skills will contribute to their academic achievement [2]. A more comprehensive approach that includes the forethought and self-reflection phases can provide more effective strategies for managing stress and promoting effective learning in workplace settings.

Although detailed intervention strategies are beyond the scope of this research, we want to provide initial guidance for practitioners. Our findings suggest that targeted interventions focusing on various individual SRL-aspects can be effective in reducing stress in the subsequent week. To achieve this, practitioners could, for instance, implement interventions specifically designed to enhance planning skills by implementing interventions that focus on structured goal-setting, effective time management, and clear task prioritization [3, 9, 112–114]. Moreover, targeted interventions or training activities designed to help learners break down complex tasks into manageable steps, set realistic deadlines, and sequence their work according to priorities can significantly enhance their planning skills [115]. The use of planning tools—such as timelines, checklists, and digital applications—can further aid in organizing tasks and ensuring systematic progress [115], thereby reducing the stress associated with unclear or overwhelming tasks. Similarly, other SRL-aspects that demonstrated a buffering effect against stress in our study—such as preparation, rehearsal, reflection, and performance approach goal orientation—could also help to reduce future stress levels in students when supported by previously established interventions. To enhance the effectiveness of SRL interventions, educators might consider utilizing SRL microanalysis [116] which provides a detailed examination of learners’ self-regulatory processes by analyzing their behaviors and strategies across the different phases of SRL [13].

Additionally, our study shows that reducing stress can positively impact a wide range of SRL-aspects (including attention, elaboration, clarification, consolidation, control, interest, effort, proactive attitude, and emotion). By creating a more supportive and less stressful learning environment through the means of implementing stress reduction interventions—such as mindfulness training and relaxation techniques [117–119], cognitive-behavioral strategies such as cognitive restructuring [120], and social support systems such as peer mentoring [121–123]— educators and practitioners can create an ecological approach that supports improvement across various areas of SRL. In addition to individual interventions, systemic strategies should also be considered to alleviate stress and support SRL. This includes avoiding overwhelming students with excessive tasks, adhering to reasonable working hours, and respecting breaks and recovery times, especially considering that many students balance both work and study commitments. Recognizing preparation (forethought phase) as well as follow-up time (self-reflection phase) as part of the working schedule rather than as additional burdens during free time could further reduce stress and enhance self-regulatory learning capacities.

Our findings suggest that while some SRL-aspects may benefit from broad stress interventions, while improving other SRL-aspects can be instrumental in mitigating stress itself. This dual focus on managing stress and enhancing SRL skills could offer a comprehensive strategy for supporting learners in navigating high-pressure work environments effectively.

## Conclusion

In general, our study demonstrated an overall negative relationship between various aspects of undergraduates’ SRL in the workplace and stress, indicating that increased stress levels are often associated with diminished SRL capabilities and that SRL helps undergraduates deal with stress. The results further emphasize the importance of dissecting the SRL process into forethought, performance, and self-reflection phases for a more nuanced understanding of how each phase interacts with stress. This nuanced understanding could provide essential information for comprehensively exploring the complexities of SRL and its interplay with stress.

## Electronic supplementary material

Below is the link to the electronic supplementary material.

Supplementary Material 1: **Additional file 1 **Structure of the Workplace Placements at the Vetmeduni Vienna. Clinics and departments included in the workplace placements at the Vetmeduni Vienna (winter term 21/22)

Supplementary Material 2: **Additional file 2 **Intraclass Correlation Coefficients (ICCs) of outcome variables. Intraclass Correlation Coefficients (ICCs) of outcome variables

## Data Availability

The dataset used and/or analyzed during the current study is available from the corresponding author upon reasonable request.
