# Supplementary material for: Workplace learning: the bidirectional relationship between stress and self-regulated learning in undergraduates
Source: BMC Med Educ. 2024 Sep 27;24:1038. doi: 10.1186/s12909-024-06021-w (PMC11429500; doi:10.1186/s12909-024-06021-w)
Supplement: Supplementary file 1 — Supplementary Material 1: Additional file 1 Structure of the Workplace Placements at the Vetmeduni Vienna. Clinics and departments included in the workplace placements at the Vetmeduni Vienna (winter term 21/22) [file 12909_2024_6021_MOESM1_ESM.docx]

## Structure of the Workplace Placements at the Vetmeduni Vienna

| Clinics and departments included in the workplace placements at the Vetmeduni Vienna (winter term 21/22). | | |
| --- | --- | --- |
| Rotation | Sub Rotation/Clinic | Department |
| Companion Animals + Equine-Medicine | Companion Animals  (5 weeks) | Anaesthesia |
|  |  | Imaging diagnostics |
|  |  | Surgery |
|  |  | Gynecology |
|  |  | Internal medicine |
|  |  | Emergency department |
|  |  |  |
|  | Equine-Medicine  (4 weeks) | Anaesthesia |
|  |  | Surgery incl. imaging diagnostics |
|  |  | Internal medicine |
|  |  | Reproduction |
|  | Equine-Medicine  (1 week nightshift) | Equine-Medicine |
|  |  |  |
| Livestock: Ruminants, Pigs, Poultry, Fish, Pathology | Ruminants  (2 weeks) | Herd management |
|  |  | VetFarm^1^ |
|  |  | VetFarm Gynecology |
|  |  | Ruminant Medicine |
|  | Pigs  (1 week) | Pigs |
|  |  | Farm Medau^2^ |
|  | Poultry/Fish  (1 week) | Poultry |
|  |  | Fish |
|  | Pathology  (1 week) | Pathology |
| *Note.* ^1^”VetFarm” is a central special facility for teaching and research at the Vetmeduni Vienna and includes different farms for cattle, pigs, sheep, and horses. ^2^”Farm Medau” is part of the VetFarm facility. | | |
