# Supplementary material for: Workplace learning: the bidirectional relationship between stress and self-regulated learning in undergraduates
Source: BMC Med Educ. 2024 Sep 27;24:1038. doi: 10.1186/s12909-024-06021-w (PMC11429500; doi:10.1186/s12909-024-06021-w)
Supplement: Supplementary file 2 — Supplementary Material 2: Additional file 2 Intraclass Correlation Coefficients (ICCs) of outcome variables. Intraclass Correlation Coefficients (ICCs) of outcome variables [file 12909_2024_6021_MOESM2_ESM.docx]

| **Additional File 2: Intraclass Correlation Coefficients (ICCs) of outcome variables.** | | |
| --- | --- | --- |
| SRL-Area | SRL-Aspect | ICC |
| Cognition | Preparation | 0.29 |
|  | Attention | 0.41 |
|  | Rehearsal | 0.39 |
|  | Elaboration | 0.41 |
|  | Clarification | 0.42 |
|  | Consolidation | 0.31 |
|  | Planning | 0.39 |
|  | Control | 0.46 |
|  | Reflection | 0.44 |
| Motivation | Expectancy of Success | 0.23 |
|  | Situational  Interest | 0.19 |
|  | Mastery  Approach | 0.26 |
|  | Performance Approach | 0.38 |
|  | Effort | 0.29 |
|  | Attention Control | 0.28 |
|  | Proactive  Attitude | 0.28 |
| Emotion | Scale^a^: Positive Emotion | 0.88 |
|  | Scale^b^: Negative Emotion | 0.08 |
| Context | Scale^c^ | 0.02 |
| Monitoring | Scale^d^ | 0.38 |
| Control | Scale^e^ | 0.41 |
| *Note*. ^a^The scale includes the emotions: pride, happiness, hope and curiosity. ^b^The scale includes the emotions anxiety, frustration, anger and sadness. ^c^The scale includes the aspects organizational framework conditions, supervisory quality and staff support. ^d^The scale includes monitoring of the cognition-, motivation-, emotion- and context area. ^e^The scale includes the controlling of the cognition-, motivation-, emotion- and context area. ICC for stress was 0.25. | | |
